# Diminished corticomotor excitability in Gulf War Illness related chronic pain symptoms; evidence from TMS study

**DOI:** 10.1038/s41598-020-75006-8

**Published:** 2020-10-28

**Authors:** Karen Lei, Alphonsa Kunnel, Valerie Metzger-Smith, Shahrokh Golshan, Jennifer Javors, Jennie Wei, Roland Lee, Michael Vaninetti, Thomas Rutledge, Albert Leung

**Affiliations:** 1 Veterans Medical Research Foundation, 3350 La Jolla Village Dr (151A), Building 13, San Diego, CA 92161 USA; 2 College of Medicine, California Northstate University, 9700 W Taron Dr, Elk Grove, CA 95757 USA; 3 Center for Pain and Headache Research, Veterans Affairs San Diego Healthcare System, 3350 La Jolla Village Dr, San Diego, CA 92161 USA; 4 School of Medicine, Department of Anesthesiology, University of California, San Diego, 9500 Gilman Dr, La Jolla, CA 92093 USA

**Keywords:** Chronic pain, Pain management, Diseases, Medical research

## Abstract

Chronic diffuse body pain is unequivocally highly prevalent in Veterans who served in the 1990–91 Persian Gulf War and diagnosed with Gulf War Illness (GWI). Diminished motor cortical excitability, as a measurement of increased resting motor threshold (RMT) with transcranial magnetic stimulation (TMS), is known to be associated with chronic pain conditions. This study compared RMT in Veterans with GWI related diffuse body pain including headache, muscle and joint pain with their military counterparts without GWI related diffuse body pain. Single pulse TMS was administered over the left motor cortex, using anatomical scans of each subject to guide the TMS coil, starting at 25% of maximum stimulator output (MSO) and increasing in steps of 2% until a motor response with a 50 µV peak to peak amplitude, defined as the RMT, was evoked at the contralateral flexor pollicis brevis muscle. RMT was then analyzed using Repeated Measures Analysis of Variance (RM-ANOVA). Veterans with GWI related chronic headaches and body pain (N = 20, all males) had a significantly (*P* < 0.001) higher average RMT (% ± SD) of 77.2% ± 16.7% compared to age and gender matched military controls (N = 20, all males), whose average was 55.6% ± 8.8%. Veterans with GWI related diffuse body pain demonstrated a state of diminished corticomotor excitability, suggesting a maladaptive supraspinal pain modulatory state. The impact of this observed supraspinal functional impairment on other GWI related symptoms and the potential use of TMS in rectifying this abnormality and providing relief for pain and co-morbid symptoms requires further investigation.

**Trial registration**: This study was registered on January 25, 2017, on ClinicalTrials.gov with the identifier: NCT03030794. Retrospectively registered. https://clinicaltrials.gov/ct2/show/NCT03030794.

## Introduction

Gulf War Illness (GWI) is a chronic multisymptomatic illness that uniquely affects military personnel of the 1990–91 Persian Gulf War. An estimated 25–32% of the 700,000 Veterans returning from theater in the Persian Gulf have exhibited a sequela of symptoms characterized by GWI. These symptoms include neurological dysfunction, gastrointestinal, respiratory, or dermatological issues as well as pain and fatigue^1,2^. Of those, chronic pain has been one of the most debilitating conditions and appears to be inseparable for those who were diagnosed with Gulf War Illness^3–5^. While the pathophysiology underlying pain symptomology has not been well defined, it is well known that chronic pain states are associated with diminished intrinsic supraspinal pain modulatory function which often presents as reduced motor cortical excitability^6–10^. On the other hand, increased motor cortical excitability is known to be associated with enhanced pain inhibition^11^. Thus, assessing the underlying motor cortical excitability in patients with GWI related diffuse body pain including headaches, muscle, and joint pain can offer a new level of understanding in the illness and potentially provide guidance for therapeutic intervention for this patient population. While it is widely accepted that chronic pain states can be associated with impaired corticomotor excitability represented by a diminished supraspinal modulatory state from traumatic or non-traumatic causes^6–8,12–14^, to the authors’ best knowledge, no study has been conducted to assess the corticomotor excitability in patients with GWI related chronic diffuse body pain including headaches, joint, and muscle pain.

Transcranial magnetic stimulation (TMS) can evoke action potentials on the cerebral cortex, and is one of the most established methods for assessing motor cortical excitability via the measurement of the resting motor threshold (RMT), defined as the minimum percentage of the maximum intensity needed to elicit a motor response demonstrated on an electromyogram^15–18^. In addition, it provides a potential therapeutic option in rectifying supraspinal neoplastic and maladaptive changes occurring in chronic pain syndromes^19–22^.

To further the understanding of the pathophysiology of pain associated with GWI, this study compared RMT in Veterans with GWI-associated chronic headaches, muscle, and joint pain, with gender and age matched controls who served in the same period and region but did not exhibit these pain symptoms, hypothesizing that cortical excitability of the motor cortex is suppressed in patients with GWI.

## Methods

Veterans who served in the Persian Gulf War I between August 1990 and July 1991 were screened and enrolled based on the study protocol approved by the Institutional Human Subject Protection Committee. All methods were carried out in accordance with the approved protocol. The study consisted of three visits as follows: (1) Informed Consent and Screening; (2) MRI Scan; and (3) Resting Motor Threshold Procedure with TMS. On average, study participants completed all three visits within 2 weeks.

### Screening and demographics

The main study inclusion criteria consisted of male or female Veterans between the ages of 18 and 65 years old who served in the Persian Gulf for at least thirty consecutive days between August 1990 and July 1991. Veterans in the Gulf War Illness-associated headache and pain group (GWV-HAP) were also required to meet the following diagnostic criteria:

1. CDC Criteria for Gulf War Illness (at least 1 symptom in 2 of the following domains)^23^
  1. Pain
  2. Fatigue
  3. Cognition and Mood
2. Kansas Criteria for Gulf War Illness (at least 3 out of the 6 following domains)^24^
  1. Pain
  2. Neurologic
  3. Fatigue
  4. GI Symptoms
  5. Respiratory Symptoms
  6. Skin Symptoms
3. International Headache Society Criteria (ICHD-3) for Migraine Headaches Without Aura^25^
  1. At least 5 headache attacks, fulfilling the following criteria i and ii:
    i. Headache attacks lasting 4–72 h
    ii. Occupied by nausea and/or photophobia and phonophobia

In addition, GWV-HAP subjects were required to have an average headache exacerbation intensity greater than or equal to 3/10 on a numerical rating scale (NRS), occurring at least three times a week and at least one of which should have lasted more than four hours in the past three months^26^. They were also required to have daily muscle pain with an intensity of greater than or equal to 3/10 on a NRS and daily joint pain with an intensity of greater than or equal to 3/10 on a NRS^26^.

Age and gender matched Veterans who met the initial study criteria of a male or female Veteran between 18 and 65 years old who served in the Persian Gulf for at least thirty consecutive days between August 1990 and July 1991, but did not meet both GWI diagnostic criteria and the pain conditions defined above for GWV-HAP group were recruited for the Gulf War Veterans (GWV) Control group. GWV Control group were gender and age-matched within one year to the GWV-HAP group.

Veterans were excluded from either GWV-HAP or GWV Control groups of the study if they had any of the following: pregnancy; history of pacemaker implant; any ferromagnetic material in the brain and/or body; history of dementia; major psychiatric diseases; life threatening diseases; presence of any chronic neuropathic pain; history of seizures; pending litigation; low back pain with mechanical origins; lack of ability to understand or speak English; history of traumatic brain injury; chronic tension or cluster headaches; or ongoing cognitive rehabilitation or treatment for Post-Traumatic Stress Disorder (PTSD).

After subjects were enrolled into the study, demographic information pertaining to age, gender, race, body mass index (BMI), and Persian Gulf War durations and duties were collected. Those who served in the 1990–91 Persian Gulf War were indicated as serving under “Gulf War I,” whereas those who served in both the 1990–91 Persian Gulf War and post-1991 Gulf War were indicated as “Both.” Time served in the Persian Gulf was reported in months. Military branches were reported as either Navy, Marines, Army, or Other, with “Other” consisting of the Coast Guard and Air Force branches. Combat status was reported based on military occupational specialty, with combat roles coded as “C” and non-combat roles coded as “NC”.

### Neuroimaging data acquisition

Neuroimaging data was acquired through magnetic resonance imaging (MRI) using a General Electric Discovery MR750 3.0T scanner. During the scan, subjects were instructed to keep the head still and padding was provided between the subject’s head and the scanner head coil to minimize head movement. Anatomical scans were obtained with magnetization prepared rapid gradient echo (MP RAGE) samplings (176 slices, T1 450, TE 3.172, TR (ms) 8.132, 256 × 256 and 1 mm slice thickness).

### Study procedure: resting motor threshold

#### Preparation

Subjects were asked to sit in a comfortable chair and relax as much as possible. Electromyography (EMG) recordings (Xltek, Oakville, Ontario, Canada) were collected through silver-silver chloride surface electrodes attached to the contralateral flexor pollicis brevis muscle and a 3 cm diameter ground electrode placed on the back of the hand. Manufacturer preinstalled software was used to collect signal with a recording time window of 200 ms, gain of 100 μV, notch filter 60 Hz, LFF 20 Hz, and HFF 10 k Hz. TMS was performed with a figure-of-eight Cool-B65 coil connected to MagPro R30 (Alpine BioMed, Fountain Valley, CA, USA).

#### TMS neuronavigation system

All anatomical MRI images were processed using BrainVoyager TMS Neuronavigation Software (Brain Innovation, Maastricht, The Netherlands), in which anatomical scans were oriented along the anterior commissure (AC) and posterior commissure (PC) plane. The transformed images were used to create 3D head and brain meshes, where head fiducial points (FDP) were marked. Utilizing the BrainVoyager ultrasound-based co-registration system via sensors attached to each subject’s face and the TMS coil, stereotaxic data was used to determine the spatial position of the TMS stimulation site in relation to each subject. A digitizing pen with ultrasound sensors was used to mark each subject’s face, which was co-registered to corresponding FDPs on a subject’s head mesh. As a result, the TMS coil was visualized in real time and space, allowing the investigator to focus the magnetic flux on a specific target region.

#### Resting motor threshold

Under the guidance of BrainVoyager Neuronavigation, a constant suprathreshold stimulus intensity was applied from the TMS coil on the left primary motor cortex (LMC) in an ascending order. Given the pain symptoms in this patient population are diffuse, the laterality of the testing side was chosen for the ease of operation and location derived from previous pain related studies. A coil holder (Alpine BioMed, Fountain Valley, CA, USA) with three joints (two of which are ball joint) was used to hold the coil in place during the assessment. A single pulse TMS was delivered to the LMC starting at 25% of maximum stimulator output (MSO) and increasing in steps of 2% until a motor response at the flexor pollicis brevis muscle of 50 µV peak to peak amplitude in five out of ten consecutive trials was evoked. Each pulse was delivered at 20–30 s apart to minimize any potential lingering effect from the preceding pulses. The maximum vertical coil-to-target distance was recorded and the horizontal distance between the center of the magnetic flux and the cortical target was kept under 5 mm during each pulse delivery^27–29^. The MSO of the MagPro R30 used in this procedure was 1850 V, and the minimum stimulus required for the evoked response was defined as the resting motor threshold (RMT)^30^. The cortical location for flexor pollicis brevis muscle was obtained by using the personalized brain mesh to trace the precentral gyrus region corresponding to the contralateral hand in the homunculus layout, which was confirmed using the EMG. All assessments were conducted in the same ambient (temperature and lighting) setting between 10 am and 2 pm during weekdays.

### Data analysis

The within subject design was used for this study. All subjects in GWV-HAP group were age and gender matched to a counterpart control group, and there was no significant difference between them on these measurements using Chi-Square for categorical data and Analysis of Variance for continuous data. Furthermore, these two groups were not significantly different on race, Gulf War period, duration, number of deployments, military branch, combat status, or BMI. The primary outcome, resting motor threshold (RMT) data, was normally distributed and analyzed using Repeated Measures Analysis of Variance (RM-ANOVA) with one within-factor of headache and pain using SPSS version 23.

### Ethics approval and consent to participate

All research subjects provided informed consent for the study. The research protocol was approved by the Veteran’s Affairs San Diego Healthcare System Institutional Review Board for Human Research Protection.

### Consent for publication

Written informed consent for publication of clinical details was obtained from the patients.

## Results

### Demographics

Forty-seven Veterans were screened based on the study protocol approved by the Institutional Human Subject Protection Committee. Out of the forty-seven Veterans, one failed the study screening, five dropped prior to study procedures due to time commitment or MRI complications, and one was excluded due to information shared after the study procedures that would exclude him or her from the study. The remaining forty gender and age-matched Veterans (20 GWV-HAP and 20 GWV Control) were enrolled in the study and their data analyzed. On average, the average age of Veterans (years old ± SD) in the GWV-HAP (N = 20, all males) group was 50.7 ± 4.2 years old, serving an average duration (months ± SD) of 7.9 ± 8.7 months in the Persian Gulf over 1.6 ± 1.2 (numbers ± SD) of deployments (Table 1). The average age of Veterans (years old ± SD) in the GWV Control group (N = 20, all males) were 50.8 ± 4.2 years old and served 8.2 ± 3.8 months in the Persian Gulf with 1.3 ± 0.6 number of deployments (Table 2). All subjects were male in both groups. Five Veterans in the GWV-HAP group served in both Gulf War I and post-1991 Gulf War periods, whereas four in the GWV Control group served in both wars. The remaining Veterans served in only Gulf War I. The GWV- HAP group consisted of twelve Caucasians, three Asians, two African-Americans, two Hispanics, and one Pacific Islander, whereas the GWV Control group consisted of eleven Caucasians, five African-Americans, two Hispanics, one Pacific Islander, and one African-American/Asian. In the GWV-HAP group, nine Veterans were in the Navy branches, seven in the Marines, three in the Army, and one in Other, compared to ten Navy, six Marine, and four Army subjects in the GWV Control group. Eight subjects in the GWV-HAP group were in Combat (C) roles, compared to three subjects in the GWV Control group. The remaining subjects were Non-Combat (NC). Body Mass Index (BMI ± SD) in the GWV-HAP group was higher at 30.3 ± 4.0 compared to the GWV Control group who had an average BMI of 29.8 ± 4.0. BMI data was missing from one subject in the GWV Control group, who was excluded from the calculation of its average.

**Table 1:** GWV-HAP Group Demographics.

Subject	Gender	Age	Race	Gulf War Period	Duration in Gulf (months)	# of Deployments	Military branch	Combat status (C/NC)	BMI
1	M	45	African American	Both	13	3	Navy	NC	25.9
2	M	46	Pacific Islander	Gulf War I	2	1	Navy	NC	35.6
3	M	46	Caucasian	Gulf War I	6	1	Navy	NC	28.8
4	M	47	Caucasian	Gulf War I	6	1	Marines	C	29.2
5	M	47	African American	Both	39	6	Marines	C	28.5
6	M	47	Hispanic	Both	16	2	Navy	NC	31.5
7	M	48	Caucasian	Gulf War I	3	1	Other	NC	31.7
8	M	48	Caucasian	Gulf War I	5	1	Navy	NC	30.2
9	M	49	Caucasian	Gulf War I	6	1	Marines	NC	31.4
10	M	49	Caucasian	Gulf War I	10	1	Marines	C	30.0
11	M	50	Caucasian	Gulf War I	2	2	Navy	C	23.0
12	M	51	Caucasian	Gulf War I	3	1	Marines	C	28.0
13	M	51	Caucasian	Gulf War I	3	1	Marines	NC	32.2
14	M	53	Hispanic	Gulf War I	8	1	Army	C	31.6
15	M	53	Caucasian	Gulf War I	4	1	Marines	C	34.9
16	M	55	Asian	Gulf War I	3	1	Army	C	32.4
17	M	55	Asian	Gulf War I	4	1	Navy	NC	23.5
18	M	56	Caucasian	Both	6	2	Navy	NC	40.1
19	M	57	Caucasian	Both	16	2	Navy	NC	27.1
20	M	60	Asian	Gulf War I	6	1	Army	NC	30.3
Average ± SD (*N* = 20)		50.7 ± 4.2			7.9 ± 8.7	1.6 ± 1.2			30.3 ± 4.0

**Table 2:** GWV Control Group Demographics.

Subject	Gender	Age	Race	Gulf War Period	Duration in Gulf (months)	# of Deployments	Military Branch	Combat Status (C/NC)	BMI
21	M	46	Hispanic	Gulf War I	4	1	Marines	NC	26.2
22	M	46	Caucasian	Gulf War I	8	1	Army	NC	26.0
23	M	46	Caucasian	Gulf War I	6	1	Marines	C	29.8
24	M	47	Caucasian	Both	17	2	Marines	C	30.0
25	M	47	African American	Gulf War I	6.5	1	Navy	NC	35.6
26	M	48	African American	Gulf War I	3	1	Navy	NC	37.0
27	M	48	Caucasian	Gulf War I	3	1	Marines	NC	37.3
28	M	48	Hispanic	Gulf War I	6	1	Army	NC	-
29	M	49	African American/Asian	Gulf War I	6	1	Marines	C	30.5
30	M	49	African American	Gulf War I	6	1	Navy	NC	29.7
31	M	49	Pacific Islander	Both	8	2	Navy	NC	27.9
32	M	51	Caucasian	Both	15	2	Navy	NC	29.0
33	M	51	Caucasian	Gulf War I	10	1	Navy	NC	26.3
34	M	53	Caucasian	Gulf War I	9	1	Army	NC	25.8
35	M	54	Caucasian	Gulf War I	5	1	Navy	NC	25.4
36	M	55	Caucasian	Gulf War I	11	1	Marines	NC	27.2
37	M	56	African American	Both	12	3	Navy	NC	28.3
38	M	56	Caucasian	Gulf War I	6	1	Navy	NC	34.0
39	M	58	Caucasian	Gulf War I	11	1	Army	NC	34.4
40	M	59	African American	Gulf War I	12	1	Navy	NC	25.7
Average ± SD (*N* = 20)		50.8 ± 4.2			8.2 ± 3.8	1.3 ± 0.6			29.8 ± 4.0

There were no significant differences found between groups in all demographic categories (see Table 3).

**Table 3:** Between Group Differences.

	Values	GWV-HAP	GWV-Control	*P* value
**Gender**	N(%)			1.000
Male		20 (100%)	20 (100%)	
Female		0 (0%)	0 (0%)	
**Age**	Avg ± SD	50.7 ± 4.2	50.8 ± 4.2	0.911
**Race**	N(%)			0.675
Caucasian		12 (60%)	11 (55%)	
African American		2 (10%)	5 (25%)	
Hispanic		2 (10%)	2 (10%)	
Pacific Islander		1 (5%)	1 (5%)	
Asian		3 (15%)	0 (0%)	
Other		0 (0%)	1 (5%)	
**Gulf War Period**	N(%)			0.705
Gulf War I		15 (75%)	16 (80%)	
Both		5 (25%)	4 (20%)	
**Duration in Gulf (months)**	Avg ± SD	7.9 ± 8.7	8.2 ± 3.8	0.933
**# of Deployments**	Avg ± SD	1.6 ± 1.2	1.3 ± 0.6	0.313
**Military Branch**	N(%)			0.736
Navy		9 (45%)	10 (50%)	
Marines		7 (35%)	6 (30%)	
Army		3 (15%)	4 (20%)	
Other		1 (5%)	0 (0%)	
**Combat Status**	N(%)			0.077
Combat		8 (40%)	3 (15%)	
Non-Combat		12 (60%)	17 (85%)	
**BMI**	Avg ± SD	30.3 ± 4.0	29.8 ± 4.0	0.695

There were no significant differences found between groups in all demographic categories where *P* = 0.05.

### Resting motor threshold

**Figure 1:**
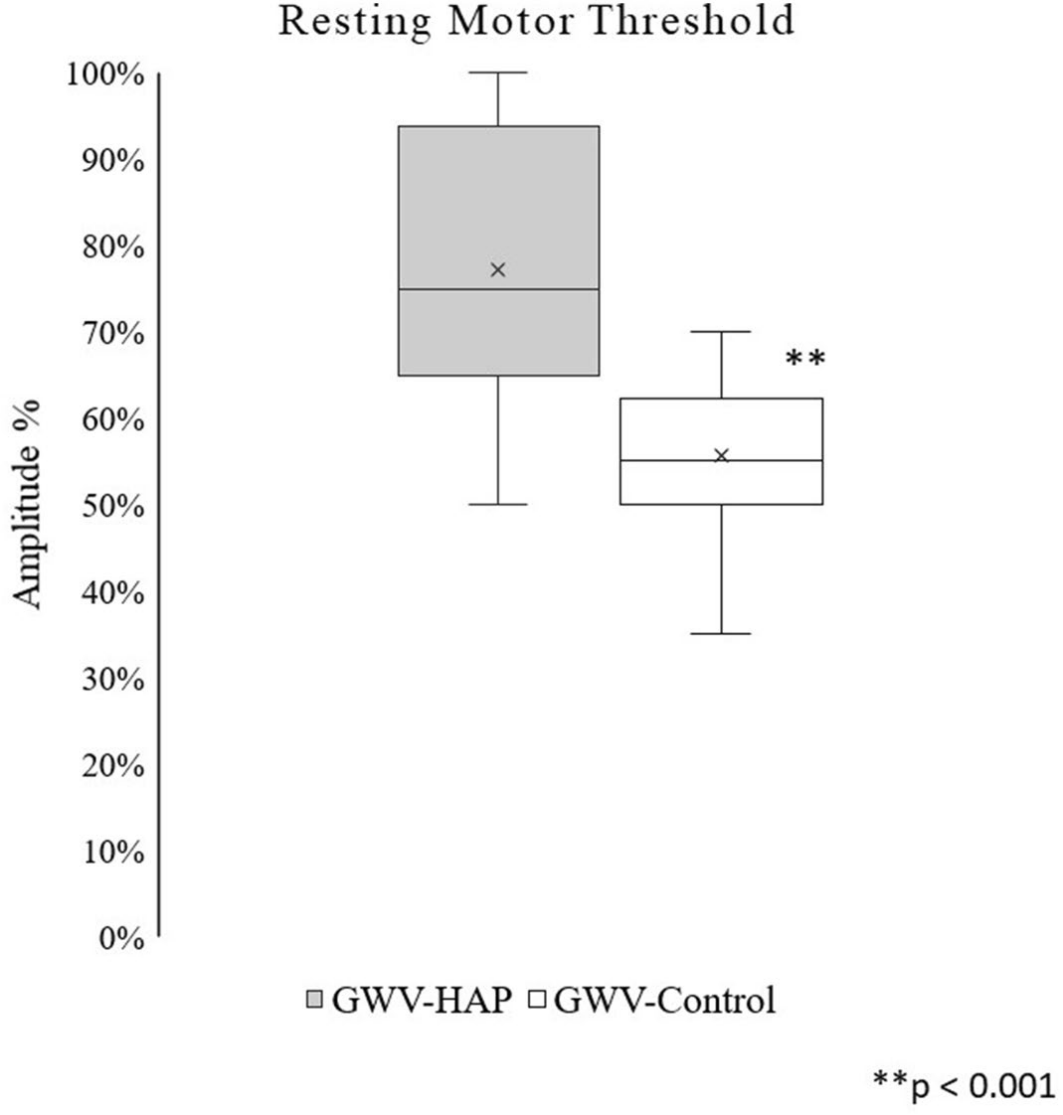
Box-and-whisker plot of the minimum value, lower quartile, median, mean, upper quartile, and the maximum value of the resting motor threshold amplitude percentages of veterans in the GWV-HAP and GWV-Control groups. Resting motor threshold was determined using electromyograph recordings on the contralateral flexor pollicis brevis muscle with the TMS coil positioned over the primary motor cortex (M1). GWV-HAP: Veterans in the Gulf War Illness-associated headache and pain group; GWV-Control: Gulf War Veterans not experiencing Gulf War Illness and associated headache and pain; **p < 0.001.

GWV-HAP subjects required a significantly higher level of stimulation to evoke a motor response with an average RMT (% ± SD) of 77.2% ± 16.7% compared to GWV Control subjects whose average RMT (% ± SD) was 55.6% ± 8.8% (f_1,19_ = 24.24, *p* < 0.001), with the GWV-HAP subjects showing a median of 75% and range from 50% to 100% and GWV Control showing a median of 55% and range from 35% to 70% (Fig. 1). In addition, there was no significant coil-to-target distance difference between the two groups with the average distance (mm ± SD) at 33.4 ± 8.3 and 33.4 ± 10.2 for GWI-HAP and GWV Control groups respectively.

### Discussion

Supraspinal pain perception consists of mainly three functional regions: (1) sensory discriminatory regions such as the primary and secondary somatosensory cortices (S1 and S2) and inferior parietal lobe (IPL); (2) affective regions such as anterior cingulate cortex (ACC) and insula (IN); and (3) modulatory regions involving motor and various regions of prefrontal cortices (PFCs)^31,32^. Additionally, the insula (IN) has been known to play a role in assessing the magnitude of pain, while the inferior parietal lobe (IPL) aids in distinguishing spatial discrimination in pain perception^33–36^. Chronic pain state is often associated with a mal-adaptation in the supraspinal pain processing, which is often accompanied with diminished modulatory functional connectivity from the prefrontal cortices with diminished motor cortex excitability as reflected by an elevated resting motor threshold^10,37^.

While chronic pain conditions are found in 60–70% of Veterans who have served in the 1990–91 Persian Gulf War in general, for those who met the diagnostic criterial of GWI, the prevalence of chronic pain is 100%^5^. Given the inseparable relation between GWI and pain, it would be infeasible to assess the illness/syndrome alone without pain.

In addition, in patients with GWI, previous diffusion tensor imaging studies revealed higher mean diffusivity and lower fractional anisotropy values along the corona radiata, corpus callosum, corticospinal tract, internal capsule, anterior and posterior thalamic radiation, fronto-occipital fasciculus, and frontal, temporal, and precentral and postcentral gyri, suggesting possible white matter reorganization or neurodegeneration^38–41^. Not only do these regions have known associations to the regulation of nociceptive input and can serve as a connection between hemispheres and cortices of the brain, they also play a role in attention, mood, fatigue, and cognition^42–48^. Thus, it will be impossible to assess the one symptom alone at a time without the others in this patient population if the focus is in understanding a common pathophysiology leading to various co-morbid symptoms.

In the current study, GWV-HAP group demonstrated an increase in RMT level compared to their non-pain Gulf War veteran counterparts. The need for a higher level of stimulation in GWV-HAP group is consistent with other chronic pain conditions caused by either direct or indirect neuronal traumas which resulted in impaired supraspinal pain modulation^49–57^. Although the pathophysiology behind the headaches and diffuse body pain in this population has not been explicitly defined, the observed functional deficit in supraspinal cortical modulatory function serves as a significant step in understanding the pathophysiology underlying the high prevalence of chronic pain states in this patient population, especially for those who meet the diagnostic criteria for GWI^58–60^.

While transcranial magnetic stimulation-evoked resting motor threshold has been demonstrated to be a reliable method for assessing cortical excitability, it may also be a viable solution for the chronic pain experienced by these Veterans. TMS is approved by the United States Food and Drug Administration for treating depression and migraine headaches^61–63^. In addition, multiple meta-analyses and panel consensus review definitively supported a high level of evidence for treating central neuropathic states, such as in the management of chronic and debilitating headaches in mild Traumatic Brain Injury patients^64,65^. High frequency (> 1 Hz) TMS works by evoking action potentials to directly excite the motor cortex and potentially regain cortical excitability, which cannot be accomplished with traditional pharmacological methods^19,20,22,66,67^. Since Gulf War Veterans with Gulf War Illness-associated headaches and pain present with a similarly increased RMT profile, it is possible they may also find relief with repetitive TMS in the form of increased pain modulatory function.

Limitations to the study include those due to an all-male study population and recruitment from a single site. Although all subjects were successfully gender and age-matched to a control group, all forty Veterans were male which is not indicative of the true Veteran population deployed to the Persian Gulf in 1990–91. While female Veterans were screened for the study, they were far fewer in number compared to male Veterans who met the criteria. This is most likely explained by the inherently male-dominated target population of 1990–91 GWV, of which only 7% of the total deployed personnel consisted of females^68^. Furthermore, 70–78% of female Veterans reported pain symptoms, meaning only a small number of female Veterans would meet the Control criteria while also living near the recruitment site^69,70^. Other reasons that may have led to this all-male result include the current distribution of female to male Veterans at the site. Since Veterans with pain required a gender and age matched healthy counterpart, it became less likely that a female was included in the study. Additionally, recruitment occurred primarily in San Diego County, which is heavily dominated by Naval and Marine bases. Future studies would benefit from recruitment from multiple sites as it would provide a study population that is more representative of the 1990–91 Persian Gulf War Veterans in terms of military branches and gender. In addition, there are inherent difficulties in conducting resting motor threshold measurements with TMS as slight variations in skull thickness, hydration, and even amount of hair among the subjects may have a minor effect on these physical parameters. However, having kept the variations in both horizontal and vertical coil-to-target distances at the minimum, the authors reckoned the impact of these individual variations should have been minimized. While circadian factors and sleep pattern may have an impact on motor cortical excitability^71^, given all RMT assessments in the current study were conducted in the same ambient setting during a 4–5 h day time window and none of the subjects have reported any sleep deprivation events prior to the assessment, the authors reckoned the potential environmental impact on the evaluation was unlikely.

In short, the observed result from the current study suggests that the high prevalence of headaches and diffuse body pain in 1990–91 Persian Gulf War Veterans, especially for those who met the diagnostic criteria of GWI, is associated with impaired cortical excitability and pain modulation. The significance of TMS in this study is two-fold: 1) it can be utilized as a tool for assessing supraspinal maladaptive state associated with the illness; and 2) it can also serve as a potential therapeutic tool in managing this multisymptomatic illness. Assessing the latter will require more well signed randomized controlled studies.

## Data Availability

The data used and/or analyzed in this study are available from the corresponding author on reasonable request.

## Abbreviations

TMS: Transcranial magnetic stimulation
GWI: Gulf War Illness
RMT: Resting motor threshold
M1: Primary motor cortex
MSO: Maximum stimulator output
GWV: Gulf War Veterans
GWV-HAP: Veterans in the Gulf War Illness-associated headache and pain group
ICHD-3: International Headache Society Criteria
NRS: Numerical rating scale
PTSD: Post-Traumatic Stress Disorder
BMI: Body mass index
C: Combat
NC: Non-Combat
MRI: Magnetic resonance imaging
MP RAGE: Magnetization prepared rapid gradient echo
EMG: Electromyography
AC: Anterior commissure
PC: Posterior commissure
FDP: Head fiducial points
LMC: Left primary motor cortex
RM-ANOVA: Repeated Measures Analysis of Variance
SSC1: Primary somatosensory cortices
SSC2: Secondary somatosensory cortices
PFCs: Prefrontal cortices
IN: Insula
IPL: Inferior parietal lobe
